# Supplementary material for: Antimicrobial and Antivirulence Activities of Carvacrol against Pathogenic Aeromonas hydrophila
Source: Microorganisms. 2022 Oct 31;10(11):2170. doi: 10.3390/microorganisms10112170 (PMC9699308; doi:10.3390/microorganisms10112170)
Supplement: Supplementary file 1 [file microorganisms-10-02170-s001.zip › Supplementary Materials.pdf]

Supplementary Materials

Table S1. Primer sequences for real-time PCR.

Primers	Gene function	Sequence (5′→3′)	Reference
ropB	Housekeeping gene	F:ACCGACGAAGTGGACTATCT<br>R:CGGCGTTCATAAAGGTGGAT	[31]
flaB	Motility and adhesion: polar flagellin B	F:CAGTCTGAACCAGACAGGTG<br>R:CAGCCATTACGTTTTGAGCC	[31]
aha	Adhesion: adhesin	F: AAGCCGTCAAGGTTACTGAC<br>R: GTCACCAGTGTTGTTGGTCT	[31]
ompA	Porin and adhesion: outer membrane protein A	F: TGGATCTGCAAGCTCGTTAC<br>R:CTACGTAGGAAGTGCGGAAC	[31]
ahp	Enzyme: serine protease	F: TCTATGCGCTGGAGTCGTTC<br>R: AGGACATGCCCACGTTGTAG	[31]
ela	Enzyme: elastase	F: TACCGCAACTGGTACAACAC<br>R:CGGAGTTCTGCTCGGTAAAG	[31]
act	Toxin: cytolytic centerotoxin	F: TCAAGGCCGATGTCAGCTAT<br>R: GTCCCACTGGTAACGAATGC	[31]
aerA	Enzyme: aerolysin	F:AGGAGATGTCAGCCTTGTAG<br>R: TTACGATACCGCCACCAACT	[63]
hly	Toxin: hemolysin	F: TCTACCTCAACGTCAACCGC<br>R: TCCGCACTATCTTGGCATCC	[31]
AhyR	Quorum system	F: TCTTGACGTGATGGGGTTGG<br>R: GGCGGTGATGAACGACAGTA	[64]

Table S2. Fractional inhibitory concentration (FIC) and fractional inhibitory concentration index (FICI) of carvacrol in association with different combinations of the antimicrobials florfenicol (FLF), enrofloxacin (ENF), doxycycline hyclate (DOH), thiamphenicol (THM), and neomycin sulfate (NES) against *Aeromonas hydrophila* NJ-35.

Test substance	MIC (μg/ml)		FIC	FICI
	Isolate	Combined		
Carvacrol−FLF				
Carvacrol	125.000	125.000	1.000	1.063[c]
FLF	1.250	0.078	0.063	
Carvacrol−ENF				
Carvacrol	125.000	125.000	1.000	1.100[c]
ENF	0.010	0.001	0.100	
Carvacrol−DOH				
Carvacrol	125.000	125.000	1.000	1.062[c]
DOH	0.625	0.039	0.062	
Carvacrol−THM				
Carvacrol	125.000	125.000	1.000	1.500[c]
THM	2.500	1.250	0.500	
Carvacrol−NES				
Carvacrol	125.000	7.813	0.063	0.563[b]
NES	20.000	10.000	0.500	

[b] Additivity; [c] Indifferent; MIC, minimum inhibitory concentration.

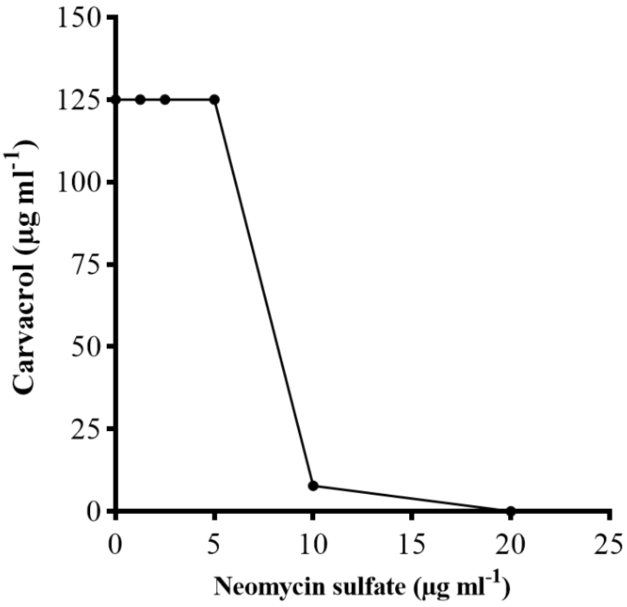

Figure S1: Isobolograms. Carvacrol combined with the neomycin sulfate against *A. hydrophila* NJ-35 (FICI = 0.563). FICI, fractional inhibitory concentration index.
